# McKittrick-Wheelock Syndrome Presenting with Acute Kidney Injury and Metabolic Alkalosis: Case Report and Narrative Review

**DOI:** 10.1155/2019/3104187

**Published:** 2019-01-31

**Authors:** Mireille Caron, Charles-Etienne Dubrûle, François Letarte, Valéry Lemelin, Alexandre Lafleur

**Affiliations:** ^1^Faculty of Medicine, Laval University, Québec City, QC, Canada; ^2^Department of Internal Medicine, CHU de Québec, Québec City, QC, Canada; ^3^Department of Surgery, CHU de Québec, Québec City, QC, Canada; ^4^Department of Gastroenterology, CHU de Québec, Québec City, QC, Canada; ^5^Faculty of Medicine, Department of Medicine, Laval University, Québec City, QC, Canada

## Abstract

A rare combination of severe volume depletion and electrolyte imbalance caused by a rectal villous adenoma is often referred to as the McKittrick-Wheelock syndrome. Patients usually seek medical care because of chronic hypersecretory diarrhea and display renal failure, metabolic acidosis, hyponatremia, and hypokalemia. We report the case of a 68-year-old woman who presented with this condition but showed unusual features such as severe hypokalemia and metabolic alkalosis, without diarrhea. She subsequently underwent transanal endoscopic microsurgery (TEMS), an innovative procedure in the management of large rectal adenomas. We also provide a narrative review of the literature on this rare entity.

## 1. Introduction

Most colorectal polyps are asymptomatic. However, a minority of adenomas may present with rectal bleeding, tenesmus, or even chronic diarrhea, which can lead to dehydration. A rare combination of severe volume depletion and electrolyte imbalance caused by a large rectal villous adenoma is often referred to as the McKittrick-Wheelock syndrome. Here we outline the case of a 68-year-old woman who developed this condition and review the literature on this topic. This report aims to increase awareness for this reversible but potentially lethal entity, especially when atypical features are present. Prior informed patient consent was obtained for publication of the case details.

## 2. Case Report

**Figure 1:**
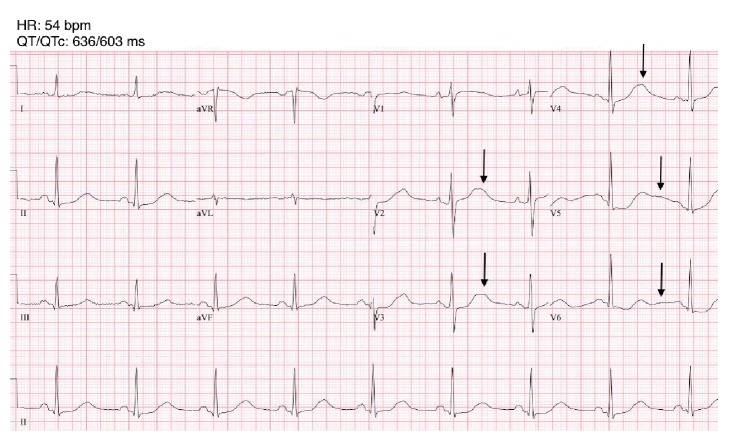
Electrocardiogram performed on first visit. The U waves are seen in precordial derivations (arrows). In V2-V3-V4, they are fused to the end of the T waves. In V5-V6, inverted T waves are followed by subtle U waves. The pseudo-prolonged QT interval actually results from the measurement of a long QU interval. HR = heart rate; bpm = beats per minute; QT = QT interval; QTc = corrected QT interval; ms = milliseconds.

A 68-year-old woman with no previous medical history presented to the Emergency Room for weakness, dizziness, and nausea of a few days duration. At presentation, blood pressure was 133/64, heart rate 51, temperature 36.8, and respiratory rate 16. An EKG showed sinus bradycardia, a prolonged QT interval, and prominent U waves (Figure 1). Blood tests revealed a creatinine of 338 *μ*mol/L. Potassium was 1,7 meq/L, sodium 120 meq/L, chloride 61 meq/L, pH 7.57, and bicarbonates 43 meq/L. Urinalysis was as follows: sodium, 6 meq/L; potassium, 28 meq/L; chloride, <10 meq/L. Serum renin and aldosterone were ordered upon admission; after a few days delay, the values came back elevated at 152 ng/L and 3000 pmol/L, respectively. An abdominal ultrasound showed normal kidneys and bladder and a moderate quantity of fluid in the rectum. A renal scintigraphy revealed bilateral moderately severe renal dysfunction, which was suggestive of acute kidney injury. Diuresis was overall preserved (682 cc over the first 24 hours) and improved after fluid resuscitation (1800 cc on day 2). The hemodynamic and electrolyte status were normalized following the administration of approximately 3.5 liters of normal saline intravenously (IV), 180 mEq of oral and 180 mEq of IV potassium chloride over the first two days. Awaiting some laboratory results, a working diagnosis of renal tubulopathy was later disproved. The patient was discharged one week later with spironolactone and potassium chloride tablets and was referred to a nephrologist to plan further investigations.

She presented two days later with a recurrence of symptoms, new-onset atrial fibrillation, and severe hyponatremia at 113 meq/L. Although the patient denied having diarrhea, a thorough questionnaire revealed a three-month history of soft stools and mucoid discharge per rectum. At digital rectal examination, a soft mass was palpated. Sigmoidoscopy revealed the presence of a large secretory villous adenoma extending from anal margin to 10 cm. This confirmed the diagnosis of the McKittrick-Wheelock syndrome (MWS). Multiple biopsies showed a tubulovillous adenoma with focal high-grade dysplasia.

**Figure 2:**
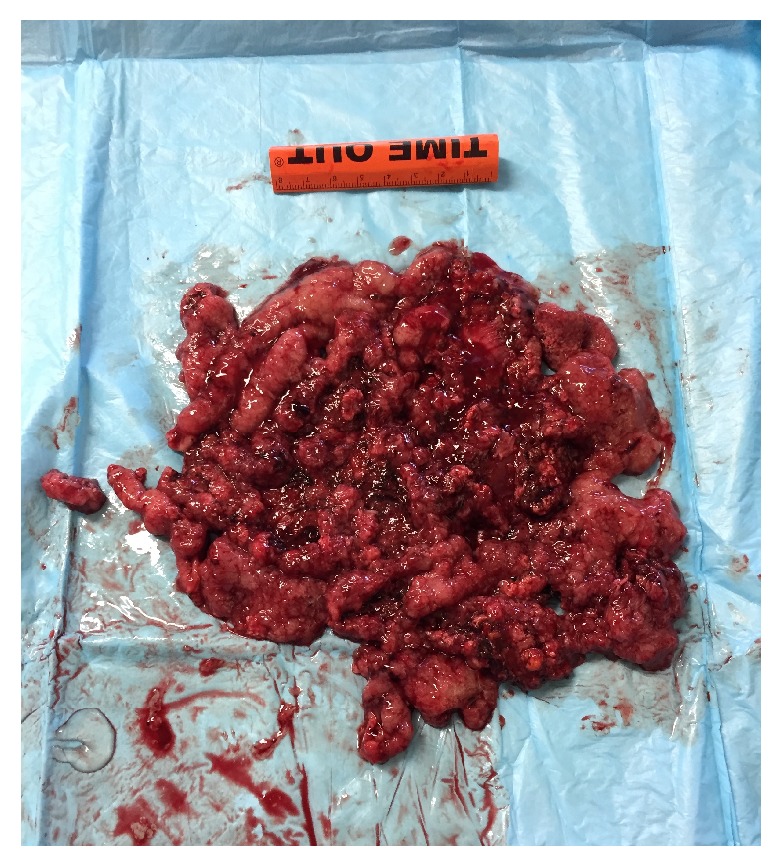
Macroscopic aspect of villous adenoma.

The patient underwent transanal endoscopic microsurgery (TEMS) successfully (Figure 2). However, atrial fibrillation recurred postoperatively and consequently she was started on low-molecular weight heparin and warfarin. Creatinine and electrolytes all normalized after surgery.  Table 1 shows the evolution of laboratory values from initial admission to postoperative day 4. She was discharged on postoperative day 6. Final pathology confirmed clear resection margins and the absence of invasive adenocarcinoma.

## 3. Discussion

A syndrome of severe volume depletion and electrolyte imbalance caused by a villous adenoma was first described by McKittrick and Wheelock in 1954 [4]. Prostaglandin E2 and cyclic AMP have both been implicated as secretagogues produced by the adenomatous tissue in cases of MWS [5].

This syndrome occurs exclusively with tumors located in the rectum; according to an early series of 48 cases, only eight were above the reach of a finger [4]. A possible explanation for this finding is that distal localization of the tumor precludes reabsorption of the secreted fluid by the colonic mucosa. Such lesions can release up to 4000 cc of clear, thin mucus containing up to 11 grams of sodium daily [4, 6].

At least 132 cases had been reported by 2013 [7]. However, very few authors have reviewed published literature on this topic [3, 8]. We conducted a first search on PubMed using “McKittrick” AND “Wheelock”, which lead to 57 results. Of those, four were irrelevant, 12 were not published in English language, one was a retrospective study, and one article was an update on a previous case.

We launched a second search using “Secretory” AND “Villous” AND “Rectum” AND “Adenoma”, which lead to 15 results. Five of them were not published in English language, and eight were already included with first search. The two remaining articles were added to the review, for a total of 41 publications and 49 MWS cases in English language.

Table 2 shows the demographic and clinical characteristics of the 49 included patients. Mean age was 63.5 years and at least 48% were women. A vast majority of patients reported nonbloody diarrhea of variable duration. When mentioned, more patients were acidotic at presentation [9–13]. Malignant pathology (adenocarcinoma) was found in 18% of examined tumors. Of interest, two patients with a history of diabetes returned to normal glucose levels after tumor resection; it was hypothesized that diabetes was likely due to secondary hyperaldosteronism [14, 15]. A majority of patients (62%) received definitive treatment with invasive surgery, such as abdominal perineal resection with colostomy or low anterior resection. However, we noticed an increasing use of minimally invasive techniques in more recent reports (18%), including TEMS, transanal minimally invasive surgery (TAMIS), endoscopic mucosal resection (EMR), and endoscopic submucosal dissection (ESD). Empiric medical treatment with indomethacin with or without octreotide was tried in five patients [1, 2, 16–18]. Indomethacin was shown to reduce diarrhea by 50% in at least one patient, but at the cost of an increase in creatinine [2]. The benefit of octreotide is unclear; its use even resulted in a 25% increase in diarrhea in one patient, when used without indomethacin [2].

One of the unique features in our case was the absence of diarrhea. Additionally, our patient presented with a predominant metabolic alkalosis; this uncommon finding was reported in only one other case [19]. Most secretory villous adenomas of the colon cause a hyperchloremic metabolic acidosis because they produce large volumes of a potassium, bicarbonate-rich fluid. Moreover, lactate accumulation and acute kidney injury may contribute to the acidosis. However, 10 to 20% of these tumors secrete chloride rather than bicarbonate, which results in metabolic alkalosis [20]. Volume depletion also leads to secondary hyperaldosteronism, enhancing hypokalemia and alkalosis. Elevated serum renin and aldosterone in our patient supported this pathophysiology. As the etiology of normotensive hypokalemic metabolic alkalosis is not always apparent from the history, a spot urine sample and a 24-hour urine collection can be helpful in such situations. In our case, the initial finding of low urinary chloride concentration pointed toward an extrarenal loss.

In conclusion, McKittrick-Wheelock syndrome is rare and potentially lethal. Acute kidney injury and metabolic disturbances are usually reversible but will quickly recur until tumor resection is performed. Diarrhea is often but not always a prominent symptom; clinicians should question specifically for rectal mucoid discharge since patients will avoid mentioning this embarrassing symptom. Minimally invasive approaches are increasingly used in the management of this condition.

## Figures and Tables

**Table 1:** Laboratory values from first admission to postoperative day 4.

	Day 0 (admission)	Day 7 (discharge)	Day 9 (readmission)	Day 14 (PO #4)
WBC count (x 10^9^/L)	11.5	13.1	26.9	7.1
Hemoglobin (g/L)	152	127	137	90
Platelets (x10^9^/L)	191	176	330	226
Creatinine (mol/L)	338	113	127	59
Sodium (meq/L)	120	128	112	136
Potassium (meq/L)	1.7	2.7	2.5	3.8
Chloride (meq/L)	61	82	63	106
pH	7.57	7.54	7.53	
Bicarbonates (meq/L)	43	35	34	
Urine sodium (meq/L)	6	<5	<5	
Urine potassium (meq/L)	28.9	42.4	21.2	
Urine chloride (meq/L)	<10	<10	<10	
Serum renin (ng/L)	152			
Serum aldosterone (pmol/L)	3000			

PO #4 = postoperative day 4; WBC = white blood cell.

**Table 2:** Demographic and clinical characteristics of patients.

Total number of patients	49
*Age*	
Mean (years)	63.5
Min (years)	26
Max (years)	84
ND	3
*Sex, N (*%)	
Female	24 (49.0)
ND	3 (6.1)
*Hemodynamic state, N (*%)	
Hypotension and/or tachycardia	16 (32.7)
Stable	3 (6.1)
ND	30 (61.2)
*Diarrhea, N (*%)	
YES	42 (85.7)
NO	1 (2.0)
ND	6 (12.2)
*Remarkable features, N (*%)	
Prolapse	3 (6.1)
Intussusception	1 (2.0)
Reversible diabetes	2 (4.1)
Infectious endocarditis (*E. Faecalis*)	1 (2.0)
Familial adenomatous polyposis syndrome	1 (2.0)
Cronkhite-Canada syndrome	1 (2.0)
*Lab tests*	
Median creatinine, *μ*mol/L (max; IQR)	359 (1440; 447.75); 21 ND
Median sodium, meq/L (min; IQR)	118 (93; 13.5); 20 ND
Median potassium, meq/L (min; IQR)	2.6 (1.3; 0.75); 16 ND
Median chloride, meq/L (min; IQR)	67 (<45; 22.5); 36 ND
Median bicarbonates, meq/L (min; max; IQR)	16 (7.2; 31; 15.6); 38 ND
Acidosis present (pH <7,35), N (%)	6 (12.2); 42 ND
Alkalosis present (pH >7,45), N (%)	1 (2.0); 42 ND
*Pathology, N (*%)	
Benign	13 (26.5)
HGD/Cis	11 (22.4)
Adenocarcinoma	9 (18.4)
Other*∗*	1 (2.0)
ND	15 (30.6)
*Management, N (*%)	
Surgery^†^	31 (63.3)
Minimally invasive surgery^†^	9 (18.4)
Medical^†^	5 (10.2)
No treatment	4 (8.2)
ND	2 (4.1)

*∗*Pathology showed a neuroendocrine tumor [1].

^†^One patient was treated medically before surgery [2]; another patient underwent transanal minimally invasive surgery (TAMIS) and then surgery one year later for recurrence [3].

ND = not determined; min = minimum value; max = maximum value; IQR = interquartile range; HGD = high grade dysplasia; Cis = carcinoma in situ.
